# Reproductive impacts and disease burden of metritis and placental retention in dairy cows: A longitudinal monitoring study in Southern Vietnam (2022–2024)

**DOI:** 10.14202/vetworld.2025.1433-1439

**Published:** 2025-06-06

**Authors:** Thuong Thi Nguyen, Lien Thi Bich Nguyen, Khang Nguyen Duong, Thuan Khanh Nguyen

**Affiliations:** 1 Department of Clinical Veterinary Sciences, Faculty of Animal Science and Veterinary Medicine, Nong Lam University, Ho Chi Minh City, Vietnam; 2 Department of Veterinary Biosciences, Faculty of Animal Science and Veterinary Medicine, Nong Lam University, Ho Chi Minh City, Gia Lai Campus, Vietnam; 3 Research and Technology Transfer Center, Nong Lam University, Ho Chi Minh City, Vietnam; 4 Faculty of Veterinary Medicine, College of Agriculture, Can Tho City, Vietnam

**Keywords:** breeding success, calf mortality, dairy cows, metritis, placental retention, reproductive disorders, skin conductance responses monitoring, Vietnam

## Abstract

**Background and Aim:**

Postpartum reproductive disorders, particularly metritis and placental retention, significantly compromise dairy herd productivity and calf viability. In Vietnam, where dairy production is expanding, limited data exist on the prevalence and consequences of such disorders under modern farm conditions. This study aimed to determine the prevalence of metritis and placental retention in dairy cows on an industrial farm in southern Vietnam from 2022 to mid-2024 and to evaluate their reproductive and neonatal health consequences.

**Materials and Methods:**

A longitudinal study was conducted on a dairy herd monitored through skin conductance responses activity sensors and DataFlow™ II software (Allflex Livestock Intelligence, MSD Animal Health Intelligence, USA). Health alerts triggered clinical examinations to identify genital infections. Metritic cows were treated using antibiotics and hormonal therapies and were monitored for recovery and subsequent breeding success. Neonatal calves were examined for respiratory and gastrointestinal disorders for 21 days post-birth. Statistical analysis was performed using Chi-square tests at a 95% confidence level.

**Results:**

Genital infections affected 23.5%–38.8% of cows annually, with metritis prevalence ranging from 7.2% to 9.8%. Placental retention remained consistent at approximately 13% across years. Quarter 2 consistently exhibited the highest incidence of reproductive disorders. Treatment success for metritis was high (85.8%–88.6%); however, post-treatment pregnancy rates declined over time (68.5% in 2022 and 54.8% in 2024). Neonatal respiratory infections (2.9%–4.4%) were more frequent than gastrointestinal infections (0.1%–0.8%), with calf mortality declining from 3.4% in 2022 to 0.7% in 2024.

**Conclusion:**

Metritis and placental retention remain prevalent challenges in Vietnamese dairy herds, adversely impacting reproductive efficiency and calf health despite high treatment efficacy. The seasonal spike in disease incidence underscores the need for tailored herd health management during hotter months. Although early detection through precision monitoring improved recovery outcomes, residual effects on fertility persisted. Strengthened periparturient care, postpartum surveillance, and colostrum management are recommended to enhance both maternal and neonatal health outcomes.

## INTRODUCTION

Health issues during the periparturient period are widely encountered in dairy farming and represent a primary cause of culling in dairy cows [1]. Repeat breeding in cattle is commonly linked to suboptimal management practices, inadequate nutrition, abnormalities of the genital tract, hormonal disturbances, and infections such as endometritis. Non-specific infections of the reproductive tract often lead to conditions such as endometritis, metritis, and pyometra, particularly following complications, such as dystocia, retained placenta (RP), or metabolic disorders such as milk fever [2, 3]. As noted by Sheldon *et al*. [4], nearly half of the cows in a herd may develop uterine infections due to postpartum immunosuppression. Galvão [5] outlined several critical risk factors for the development of metritis and endometritis, including parity, twinning, abortion, dystocia, RP, stillbirth, male offspring, ketosis, and hypocalcemia. RP, a frequent postpartum issue, is currently classified as a metabolic disorder emerging during the transition period [6, 7]. In dairy cattle, RP often predisposes animals to bacterial infections, systemic illness, and reduced milk production, collectively causing significant economic losses for producers [8, 9]. The incidence of placental retention exhibits notable variability across different countries, herds, and parity levels, with reported rates ranging from 4.0% to 16.1% per calving in South Korea and approximately 7.8% in various parity groups of dairy cows in the United States [10, 11]. Hence, effective strategies for the control of metritis and placental retention are crucial to ensure optimal health and productivity within dairy herds.

Stillbirths have been associated with an increased risk of metritis and placental retention; however, they may also be linked to a decreased probability of ovulatory infertility and fertilization failure [12]. Maternal stress during late gestation can negatively affect colostrum production and fetal gastrointestinal development, impairing the calf’s ability to absorb immune and nutritional components from colostrum. This ultimately hinders the establishment of a functional post-natal mucosal immune system essential for effective digestion and nutrient uptake [13]. According to Birmachu and Hunde [14], reproductive disorders such as mastitis, abortion, retained fetal membranes, vaginal prolapse, dystocia, anestrus, repeat breeding, endometritis, and stillbirth were highly prevalent among dairy cows in Ethiopia. The study also reported a calf mortality rate of 42.9%, with the majority of deaths occurring within the 1^st^ month of life. These findings indicate a strong relationship between maternal reproductive health and the survival and well-being of neonatal calves.

Despite growing attention to reproductive health management in dairy cattle, limited longitudinal studies have been conducted in Southeast Asia – particularly in Vietnam – where the dairy industry is rapidly expanding. Existing literature has primarily focused on reproductive disorders in Western and African settings, with sparse epidemiological data available for Vietnamese herds operating under intensive production systems. Moreover, most previous studies address reproductive disorders such as metritis and placental retention in isolation, without integrating their longitudinal effects on breeding efficiency and neonatal calf outcomes. In addition, the role of seasonal variations and their impact on the incidence of uterine infections in tropical climates remains underexplored. There is also a lack of field-based evidence assessing the practical application of precision livestock monitoring tools, such as skin conductance responses (SCR) systems, in identifying and managing reproductive diseases within large-scale dairy operations. Addressing this gap is essential to inform region-specific reproductive health strategies and improve overall herd productivity in Vietnam and comparable agro-ecological contexts.

This study aimed to investigate the prevalence and temporal distribution of postpartum reproductive disorders, particularly metritis and placental retention, in a commercial dairy herd in Southern Vietnam over a 2.5-year period (2022-mid-2024). Furthermore, the research evaluated the clinical recovery rates and post-treatment reproductive performance of cows diagnosed with metritis, as well as the incidence of respiratory and gastrointestinal infections in their newborn calves. By employing automated monitoring systems and clinical diagnostics, the study sought to provide integrated insights into how maternal reproductive health affects both breeding outcomes and neonatal viability. The findings are intended to support evidence-based reproductive management practices and enhance disease control protocols to reduce economic losses in tropical dairy systems.

## MATERIALS AND METHODS

### Ethical approval

All procedures involving animals were reviewed and approved by the Animal Welfare Committee of Nong Lam University, Ho Chi Minh City, Vietnam.

### Study period and location

This study was conducted from January 2022 to June 2024 in an industrial milk company in Tay Ninh province in Southern Vietnam.

### Housing and management conditions

The dairy cows were maintained in a free-stall barn equipped with automated cooling systems and mist sprayers, which operated for 3 min with 20-s bursts at feeding stations. Manure was continuously removed and deposited into designated collection pits. Fresh, clean water was provided *ad libitum*. Cows were fed a formulated total mixed ration tailored to their lactation and gestation stages. Milking was conducted 3 times daily at 05:00, 13:00, and 21:00 h.

### Study population and monitoring period

The study was conducted on a commercial dairy farm in Southern Vietnam. A total of 3,747 cows were observed in 2022, 3,820 in 2023, and 2,075 during the first half of 2024. Each cow was fitted with an SCR neck-mounted chip starting at approximately 12 months of age to monitor rumination and activity levels. Data were recorded bi-hourly and transmitted automatically to the DataFlow™ system (Allflex Livestock Intelligence, MSD Animal Health Intelligence, USA) every 20 min through barn-mounted antennas. A health index score (HIS) below 75 units triggered health alerts.

### Clinical evaluation of cows and calves

Cows flagged by the SCR system were subjected to physical examination by trained veterinarians. The HIS is generated by a proprietary algorithm developed by SCR Dairy, quantifying deviations in rumination and locomotion over time, with lower scores indicating compromised health. Clinical diagnoses were used to validate these alerts.

Newborn calves were examined twice daily for 21 consecutive days postpartum. Respiratory conditions were identified by symptoms such as elevated temperature, nasal discharge, and labored breathing, while gastrointestinal disorders were assessed through indicators such as abdominal pain, diarrhea, and vomiting. Diagnostic criteria followed those outlined by Kasa *et al*. [15] and Oliveira *et al*. [16].

### Treatment protocol for metritis

Cows diagnosed with clinical metritis received standardized veterinary treatment. Antibiotics and anti-inflammatory agents included intramuscular injections of ceftiofur (100 mg/mL) and ketoprofen (100 mg/mL) for 5 consecutive days. Hormonal therapy included intramuscular administration of gonadotropin (50 μg/mL), cloprostenol (87.5 μg/mL), and Vitamin AD3E (800,000 IU of Vitamin A, 40,000 IU of Vitamin D3, and 20 mg of Vitamin E) at 10-day intervals for a total of three administrations. Recovery was evaluated based on clinical reassessment 10 days after the completion of treatment.

### Assessment of breeding performance

Following clinical recovery, cows that showed no signs of metritis were selected for mating. Pregnancy status was determined through veterinary examination 21 days post-insemination. Successful conception was recorded as a positive treatment outcome. Cows that were not pregnant after the first insemination underwent a second mating, and pregnancy was reassessed 21 days later. Failure to conceive after two attempts classified the cow as a breeding failure.

### Statistical analysis

Health and breeding data collected through the SCR system and DataFlow™ II software (Allflex Livestock Intelligence) were analyzed using Minitab version 17.0. Chi-square tests were performed at a 95% confidence level to determine statistical significance.

## RESULTS

### Prevalence of genital infections and placental retention

As illustrated in Table 1, the prevalence of genital tract infections among dairy cows was notably high throughout the study period, reaching its peak in 2022 at 38.8%, followed by 32.1% in 2023 and 23.5% in mid-2024. This downward trend was statistically significant (p < 0.05). In contrast, the annual prevalence of placental retention remained relatively stable, with rates of 12.9% in 2022, 13.0% in 2023, and 13.6% in mid-2024, showing no significant interannual differences. Notably, Quarter 2 of each year consistently exhibited the highest rates of both genital infections and placental retention compared to other quarters.

**Table 1:** Reproductive diseases in dairy cows from 2022 to mid-2024.

Year	No. of examined samples from pregnant cows	Diseases after bearing (%)	

		Placental retention	Genital infection
2022	3,747	485 (12.9)	1,452 (38.8)
Quarter 1	873	138 (15.8)	406 (46.5)
Quarter 2	1,024	145 (14.2)	397 (38.8)
Quarter 3	846	121 (14.3)	316 (37.4)
Quarter 4	1,004	81 (7.9)	333 (33.2)
2023	3,820	497 (13.0)	1,227 (32.1)
Quarter 1	803	83 (10.3)	311 (35.7)
Quarter 2	1,154	164 (14.2)	403 (34.9)
Quarter 3	856	139 (16.2)	236 (27.6)
Quarter 4	1,007	111 (11.0)	277 (27.5)
2024	2,075	282 (13.6)	488 (23.5)
Quarter 1	992	110 (11.1)	204 (20.6)
Quarter 2	1,083	172 (15.9)	284 (26.2)

### Incidence and seasonal distribution of metritis

According to Table 2, metritis accounted for a substantial proportion of genital infections, with the highest annual incidence observed in 2023 (9.8%). However, no statistically significant difference was found when comparing the metritis rates between 2022 (7.3%) and mid-2024 (7.2%), nor between 2023 (9.8%) and mid-2024 (7.2%). Seasonally, the prevalence of metritis was higher during Quarters 2 and 3 across all observed years.

**Table 2:** Rate of metritis in cows after breeding from 2022 to mid-2024.

Year	No. of examined samples from cows	No. of metritis cows	Percentage
2022	1,452	106	7.3
Quarter 1	406	34	8.4
Quarter 2	397	31	7.8
Quarter 3	316	29	9.2
Quarter 4	333	12	3.6
2023	1,227	120	9.8
Quarter 1	311	28	9.0
Quarter 2	403	42	10.4
Quarter 3	236	36	15.3
Quarter 4	277	14	5.1
2024	488	35	7.2
Quarter 1	204	10	4.9
Quarter 2	284	25	8.8

### Health outcomes in newborn calves

Table 3 presents data on neonatal infections. Respiratory infections consistently exceeded gastrointestinal infections in prevalence each year. The rate of respiratory infections remained statistically similar between 2022 (2.9%) and mid-2024 (3.5%), as well as between 2023 (4.4%) and mid-2024 (3.5%). Conversely, gastrointestinal infections decreased over time, from 0.8% in 2022 and 0.6% in 2023 to 0.1% in mid-2024. Calf mortality mirrored this trend, declining from 3.4% in 2022 to 1.9% in 2023, and further to 0.7% in mid-2024.

**Table 3:** Diseases present in newborn calves from 2022 to mid-2024.

Year	No. of examined calves	Diseases after birth (%)		

		Respiratory infection	Gastrointestinal infection	Death
2022	3,747	110 (2.9)	30 (0.8)	126 (3.4)
Quarter 1	873	23 (2.6)	4 (0.5)	21 (2.4)
Quarter 2	1,024	36 (3.5)	5 (0.5)	37 (3.6)
Quarter 3	846	22 (2.6)	9 (1.1)	23 (2.7)
Quarter 4	1,004	29 (2.9)	12 (1.2)	45 (4.5)
2023	3,820	169 (4.4)	24 (0.6)	74 (1.9)
Quarter 1	803	22 (2.7)	4 (0.5)	18 (2.2)
Quarter 2	1,154	72 (6.2)	13 (1.1)	22 (1.9)
Quarter 3	856	50 (5.8)	3 (0.4)	21 (2.5)
Quarter 4	1,007	25 (2.5)	4 (0.4)	13 (1.3)
2024	2,075	73 (3.5)	3 (0.1)	16 (0.7)
Quarter 1	992	22 (2.2)	2 (0.2)	6 (0.6)
Quarter 2	1,083	51 (4.7)	1 (0.1)	10 (0.9)

### Effectiveness of metritis treatment

Treatment outcomes for cows diagnosed with metritis are summarized in Table 4. Recovery rates remained consistently high, with 86.8% in 2022, 85.8% in 2023, and 88.6% in mid-2024, showing no statistically significant variation across years.

**Table 4:** Effectiveness of treatment for metritis in cows.

Year	No. of metritis cows	No. of recovered cows	Percentage
2022	106	92	86.8
Quarter 1	34	29	85.3
Quarter 2	31	27	87.1
Quarter 3	29	25	86.2
Quarter 4	12	11	91.7
2023	120	103	85.8
Quarter 1	28	24	85.7
Quarter 2	42	36	85.7
Quarter 3	36	31	86.1
Quarter 4	14	12	85.7
2024	35	31	88.6
Quarter 1	10	9	90.0
Quarter 2	25	22	88.0

### Post-treatment breeding performance

As shown in Table 5, the post-treatment breeding performance of recovered cows was relatively stable over time. The proportion of cows that conceived after treatment was 68.5% in 2022, 67.0% in 2023, and 54.8% in mid-2024, with no statistically significant differences among the years.

**Table 5:** The breeding effectiveness of cows in the next mating after treatment.

Year	No. of cows	No. of pregnant cows	Percentage
2022	92	63	68.5
2023	103	69	67.0
2024	31	17	54.8

## DISCUSSION

This study aimed to evaluate the prevalence, treatment outcomes, and reproductive consequences of metritis and related genital infections in dairy cows, along with associated health outcomes in neonatal calves on a commercial farm in Southern Vietnam.

### Incidence and risk factors of postpartum genital infections

Postpartum genital infections were identified as a major health concern on the studied dairy farms. The elevated infection rates observed suggest inadequacies in farm hygiene and reproductive management practices. The incidence of placental retention was also notably high, indicating that uterine health challenges were widespread.

The bovine reproductive tract microbiota is a developing field of research, with emerging evidence showing that microbial communities differ significantly between healthy cows and those with uterine diseases such as metritis and endometritis [17]. These conditions are often associated with anaerobic and fastidious bacteria, suggesting a complex pathogenesis. Herd-level risk factors such as stillbirths, twin births, and dystocia – especially dystocia – have been strongly linked to RP, which in turn predisposes animals to endometritis and infertility [7, 18].

Advanced age and parity were found to compromise immune competence in high-yielding cows, increasing their vulnerability to postpartum infections. In addition, calcium imbalance during the transition period – common in older, high-producing animals – contributes to the development of hypocalcemia and RP [19, 20]. Seasonal variations in environmental microbiota, particularly during Quarter 2 (summer), may partially explain the higher incidence of genital infections. According to Nguyen *et al*. [21], microbiota shifts due to environmental factors such as bedding and dust remain poorly understood, though the use of misting fans may exacerbate microbial exposure during warmer months.

### Pathophysiological impact of metritis

Metritis was frequently diagnosed in postpartum cows with concurrent genital infections. It may induce long-term changes in the reproductive tract, leading to subfertility due to elevated uterine lipopolysaccharide levels, extended luteal phases, and suppressed endometrial immune responses [22]. Its prevention is challenging due to multifactorial etiologies including age, parity, seasonality, environmental stress, calving trauma, and RP [23]. Cows experiencing assisted parturition face increased bacterial exposure, compounding the risk of metritis and secondary infections [1].

Globally, metritis is reported in 20%–40% of dairy cows and negatively affects productivity, fertility, and longevity [24]. Abebe *et al*. [25] found that 41.6% of cows in Ethiopia exhibited at least one reproductive disorder, with metritis ranking among the top concerns. These findings highlight the need for proactive management of reproductive infections to minimize economic losses and safeguard animal health.

### Neonatal health consequences

Calves born to affected cows frequently suffered from respiratory or gastrointestinal infections and exhibited high mortality rates. Neonatal calf diarrhea, or scours, is a leading cause of morbidity and mortality, typically resulting from impaired intestinal absorption due to a range of pathogens [15]. Respiratory diseases, especially bovine respiratory disease, are also prevalent in rearing units and feedlots, where factors such as poor nutrition, transport stress, and environmental exposure weaken the immune response [16].

These findings emphasize the importance of maternal health on neonatal outcomes. The insufficient development of immune function in neonates – exacerbated by poor colostrum transfer – limits resistance to pathogens. A better understanding of bovine immunophysiology and the implementation of early nutritional and immunological interventions are essential [26].

### Nutritional and immunological interventions

Ensuring adequate colostrum intake within the 1^st^ week of life and tailoring feeding programs to physiological stages are critical to building immune competence. Maternal stress and chronic undernutrition impair immune status, thereby increasing the risk of postpartum infections such as mastitis and metritis, which ultimately reduce reproductive efficiency and increase financial losses [27].

### Treatment success and monitoring through precision tools

The high treatment success rate for metritis in this study significantly contributed to mitigating herd-level losses. The use of SCR health monitoring systems enabled early detection, timely intervention, and improved recovery outcomes. Delayed or unsuccessful treatment can lead to decreased milk yield, reduced fertility, and increased culling [28]. As highlighted by Merenda *et al*. [29], precision livestock technologies offer targeted solutions for identifying at-risk animals, improving treatment decisions, and enhancing herd-level productivity.

### Post-treatment fertility remains suboptimal

Despite clinical recovery, cows previously affected by metritis showed reduced breeding performance. Postpartum infections and uterine inflammation can persistently disrupt ovarian and endometrial function, adversely affecting oocyte quality, embryo development, and conception rates for several months [30]. Kumari *et al*. [31] found that metritis significantly influences reproductive traits, including extended calving intervals and an increased number of services per conception.

Furthermore, abnormal calving events, particularly RP, are strongly associated with prolonged open days and inter-calving intervals. As Thulasiraman *et al*. [32] noted, uterine infections impair the endocrine axis – specifically the hypothalamic-pituitary-ovarian axis – resulting in compromised reproductive function and increased culling rates.

## CONCLUSION

This longitudinal study revealed a high incidence of postpartum genital infections, particularly metritis (7.2%–9.8%) and placental retention (~13%) in dairy cows from a commercial farm in Southern Vietnam between 2022 and mid-2024. The infections were most prominent during Quarter 2 (summer), with metritis emerging as a key factor contributing to reduced reproductive efficiency. Despite high treatment success rates ranging from 85.8% to 88.6% following standardized veterinary protocols, post-treatment breeding performance remained suboptimal, as evidenced by a decline in conception rates from 68.5% in 2022 to 54.8% in 2024. In addition, respiratory infections were more frequent than gastrointestinal disorders among neonatal calves, although calf mortality significantly decreased from 3.4% to 0.7% over the study period.

The integration of automated SCR health monitoring systems facilitated early detection of metritis, enabling prompt intervention and improved recovery outcomes. These findings underscore the clinical value of precision livestock technologies in enhancing herd-level health surveillance and informing targeted veterinary responses. Concurrent postpartum monitoring of both dams and their offspring proved crucial for minimizing reproductive losses and promoting calf survival.

Among the strengths of this study are its longitudinal design, real-time monitoring capabilities through the SCR system, and the combined assessment of reproductive health and neonatal outcomes, offering a comprehensive view of maternal-offspring dynamics in tropical dairy production. However, the study’s findings are limited by its single-farm setting, which may not reflect conditions in smaller or differently managed herds. In addition, the study did not assess genetic, nutritional, or environmental microbial influences, nor did it track long-term reproductive or lactational outcomes beyond initial recovery and conception rates.

Future research should include multicenter trials across diverse Vietnamese farming systems and climatic conditions to validate these findings. Investigations into the bovine reproductive tract microbiota and its relationship with disease progression and treatment outcomes are warranted. Longitudinal monitoring of calves born to infected dams may also provide critical insights into intergenerational health impacts, and the incorporation of nutritional and immunological biomarkers could further refine herd health management strategies.

Genital tract infections – particularly metritis and placental retention – pose significant threats to dairy productivity in tropical climates. While precision health monitoring enhances clinical management and early intervention, sustained improvements in preventive care, seasonal risk mitigation, and maternal-neonatal health integration are essential. A data-driven, comprehensive approach to reproductive health will be instrumental in ensuring long-term productivity, animal welfare, and economic sustainability in dairy systems under evolving environmental and production pressures.

## AUTHORS’ CONTRIBUTIONS

TTN and TKN: Conceptualized, designed, and supervised the study. TTN and LTBN: Collected samples and conducted experiments. TTN, KND, and TKN: Analyzed and interpreted the generated data. TTN, KND, TKN, and LTBN: Drafted, reviewed, and revised the manuscript. All authors have read and approved the final manuscript.
